# Targeting a Dynamic Protein–Protein Interaction: Fragment Screening against the Malaria Myosin A Motor Complex

**DOI:** 10.1002/cmdc.201402357

**Published:** 2014-11-03

**Authors:** Christopher H Douse, Nina Vrielink, Zhang Wenlin, Ernesto Cota, Edward W Tate

**Affiliations:** [a] Department of Chemistry, Imperial College London, South KensingtonLondon SW7 2AZ (UK) E-mail: christopher.douse@googlemail.come.tate@imperial.ac.uk; [b] Centre for Structural Biology, Department of Life Sciences, Imperial College LondonSouth Kensington, London SW7 2AZ (UK); [c] Institute of Chemical Biology, Imperial College LondonSouth Kensington, London SW7 2AZ (UK)

**Keywords:** differential scanning fluorimetry, fragment screening, malaria, myosins, NMR, protein–protein interactions

## Abstract

Motility is a vital feature of the complex life cycle of *Plasmodium falciparum*, the apicomplexan parasite that causes human malaria. Processes such as host cell invasion are thought to be powered by a conserved actomyosin motor (containing myosin A or myoA), correct localization of which is dependent on a tight interaction with myosin A tail domain interacting protein (MTIP) at the inner membrane of the parasite. Although disruption of this protein–protein interaction represents an attractive means to investigate the putative roles of myoA-based motility and to inhibit the parasitic life cycle, no small molecules have been identified that bind to MTIP. Furthermore, it has not been possible to obtain a crystal structure of the free protein, which is highly dynamic and unstable in the absence of its natural myoA tail partner. Herein we report the de novo identification of the first molecules that bind to and stabilize MTIP via a fragment-based, integrated biophysical approach and structural investigations to examine the binding modes of hit compounds. The challenges of targeting such a dynamic system with traditional fragment screening workflows are addressed throughout.

## Introduction

There is a growing recognition that protein–protein interaction (PPI) hotspots may be targeted by small molecules, and fragment-based approaches have had success in this area.[1] When the target is a PPI, in vitro fragment selection relies on a series of specialized biophysical experiments to detect the weak interactions typically observed between fragments and proteins. With this in mind, Abell and co-workers recently suggested a three-stage cascade of experiments suitable for an academic laboratory, as follows: 1) screening of the initial library by differential scanning fluorimetry (DSF), 2) validation of hits by ligand-observed NMR spectroscopy, and 3) characterization of binding affinity and mode by isothermal titration calorimetry (ITC) and X-ray crystallography, respectively.[2] In particular, high-resolution co-crystal structures of bound hits are usually essential to inform subsequent elaboration by chemical synthesis.[3] However, the implementation of biophysical assays is dependent on the target in question.

**Figure 1:**
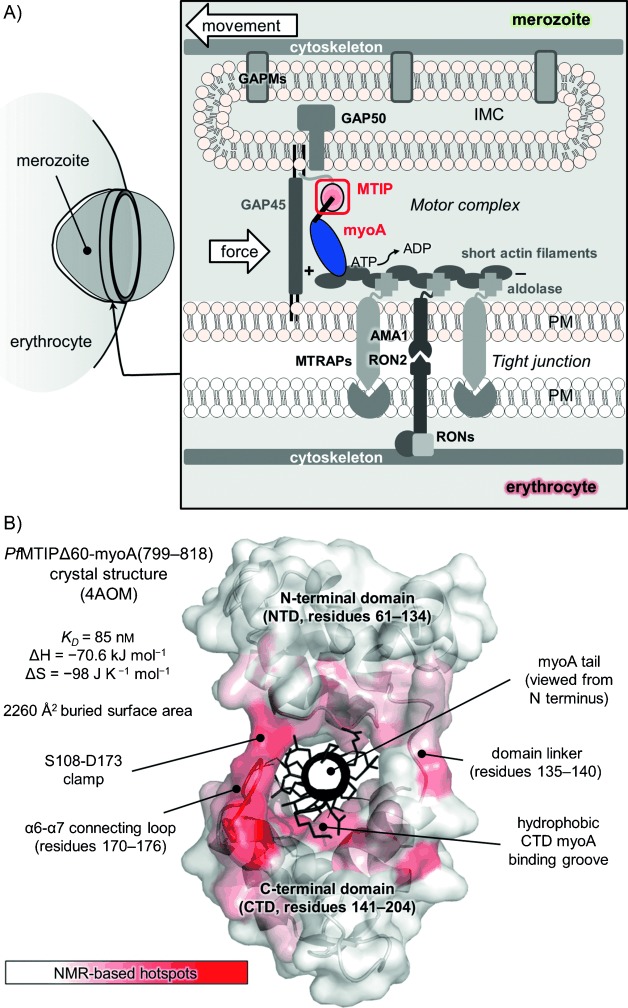
A) Linear model of the *Plasmodium* motor complex and tight junction in the context of putative roles in erythrocyte invasion by blood stage parasites (merozoites). A number of the key proteins and protein–protein interactions in this system are shown. MTRAP=merozoite-specific thrombospondin-related anonymous protein, AMA1=apical membrane antigen 1, GAP(M)=gliding-associated protein (with multiple membrane spans), RON=rhoptry neck protein, MTIP=myosin A tail domain interacting protein, IMC=inner membrane complex, PM=plasma membrane. Co-/post-translational lipid attachments are indicated on MTIP and GAP45 with black lines. B) Structural and thermodynamic features of the wild-type MTIP–myoA tail interaction.[14] Shown are the annotated crystal structure (*Pf*MTIPΔ60 shown as white cartoon and white surface, shaded by a white–red ramp according to chemical shift perturbations induced by binding to *Pf*myoA(799–818), which is shown as black cartoon and sticks) and thermodynamic parameters according to ITC experiments.

We sought to employ a fragment-based approach toward targeting the interaction between the C-terminal “tail” of *Plasmodium falciparum* myosin A (*Pf*myoA) and binding partner myoA tail domain interacting protein (*Pf*MTIP). This PPI is thought to be central to the malaria parasite’s ability to glide and invade host cells in a defined, directional manner, as correct localization of the myoA motor protein depends on its interaction with MTIP (called myosin light chain 1, MLC1, in other apicomplexan parasites such as *Toxoplasma gondii*).[4] In the prevailing model of red blood cell invasion by *Plasmodium* (Figure 1 A),[5] myoA is anchored to a “membranous network of flattened vesicles”[6] termed the inner membrane complex (IMC) via interaction with MTIP, which itself is thought to interact with “gliding-associated proteins” (GAP45 and GAP50) and is palmitoylated at its N terminus, enabling IMC attachment.[7] The action of the myoA motor is coupled to ATP hydrolysis; the force produced enables the myosin head domain to tread along short actin filaments toward the barbed (+) end. This is thought to translate into overall movement of the parasite via another set of PPIs. Tetrameric aldolase has been implicated in joining the filament to the so-called “tight junction” via the cytoplasmic tails of thrombospondin-related anonymous protein (TRAP) adhesins[8] and apical membrane antigen 1 (AMA1); the latter protein interacts with rhoptry neck protein 2 (RON2), which is inserted into the host cell plasma membrane.[9] Peptidic and, more recently, small-molecule inhibitors of the AMA1–RON2 interaction have been shown to result in a block of parasite invasion, suggesting that PPIs involved in the assembly may represent new antimalarial drug targets.[10]

We and others have developed peptidic inhibitors of the *Pf*MTIP–myoA complex by mimicking the C-terminal tail of myoA, which adopts a helical conformation and is “clamped” by two domains of MTIP (N- and C-terminal domains, NTD and CTD), burying >2000 Å^2^ of solvent-exposed protein surface upon complex formation.[11] However, no small molecules have been shown experimentally to bind to MTIP. An in silico docking study was conducted by Kortagere et al. using the non-physiological, “extended” crystal structure of *P. knowlesi* MTIP.[12] Although several of the hit compounds from this screen inhibited parasite growth with EC_50_ values on the order of 10^−7^ m, and a subset perturbed gliding motility of *Plasmodium* liver stages, all the hit compounds were highly hydrophobic, which hampered attempts to obtain biophysical evidence for MTIP binding. Furthermore, none of the inhibitors formed hydrogen bonds with MTIP residues in the docked protein–ligand complexes, suggesting that specificity of the molecules for the intended target may be low. Recently, a small-molecule inhibitor of *T. gondii* motility and invasion called tachypleginA-2 was shown to target homologous *Tg*MLC1, covalently modifying a cysteine in the disordered N-terminal extension of the protein.[13] This cysteine is not conserved in *Plasmodium* MTIPs, so the mode of action is unlikely to apply to malaria. Nonetheless, the latter work suggests that modulation of MTIP with small molecules may be disruptive to the parasite.

In our hands it has not been possible to crystallize the dynamic myoA binding construct of *Pf*MTIP (residues 61–204, herein termed *Pf*MTIPΔ60) in the absence of a myoA tail peptide; according to a 2013 report by Khamrui et al., this has also been the case for full-length *Pf*MTIP and the CTD in isolation.[15] Encouragingly, the authors were able to use a nanobody as a crystallization chaperone and to obtain a high-resolution structure of the CTD. However, the accessibility of the myoA binding groove is altered substantially by contacts between the nanobody and the MTIP construct, which may compromise subsequent efforts to discover effective small-molecule ligands. We have taken a different approach by studying isotopically labeled *Pf*MTIPΔ60 (containing both MTIP domains) in solution using multidimensional NMR techniques, and recently reported assignments of the ^1^H,^15^N-HSQC spectra of both the “free” and “bound” states, along with a detailed analysis of the conformational dynamics and thermodynamics of the system.[14] It was found that the free protein is highly flexible on a range of timescales from millisecond to sub-nanosecond exchange processes, which may explain the difficulty in crystallizing the protein in isolation. Our analysis also included the mapping of MTIP–myoA interaction hotspots from chemical shift perturbations (CSPs) upon clamping of the myoA tail (Figure 1 B). Interestingly, we found that a phosphomimetic mutation of Ser 108 in the NTD caused a pronounced structural change in the complex and a substantial decrease in affinity for myoA, even though this residue has been shown not to be in direct contact with any residue in the myoA tail. We concluded that the intramolecular clamp between Ser 108 and Asp 173 (from the CTD) is broken upon such a modification. This observation was primarily of biological interest, as the residue has been annotated as a phosphorylation site in cultured blood-stage malaria parasites,[16] but was also encouraging in relation to ligand discovery; small molecules will fail to span the large buried PPI surface area, but our analysis suggested that breaking individual hotspot interactions such as the Ser 108–Asp 173 clamp may nonetheless be inhibitory.

## Results and Discussion

### Initial screening by DSF

With the structural and dynamic features of free MTIP in mind, and given the lack of small-molecule ligands for the protein, we used differential scanning fluorimetry (DSF) to screen a library of 500 fragments for the ability to stabilize *Pf*MTIPΔ60 with respect to thermal unfolding. We have previously shown that the protein is thermally unstable in isolation, giving a melting temperature (*T*_m_) of 38.0 °C. It was also notable that the melting transition is rather shallow, interpreted as being due to extensive conformational flexibility, also reflected in NMR and ITC data.[14].

The error associated with the DSF screen was estimated by taking the standard deviation of the control sample *T*_m_ values, that is, the negative controls (5 μm *Pf*MTIPΔ60 + 1 % DMSO) and the positive controls (5 μm *Pf*MTIPΔ60 + 200 μm *Pf*myoA tail peptide). Across the 48 measurements of these samples, the standard deviations for these measurements were 0.43 and 0.52. Using a two-standard-deviation cutoff reported recently for the screen against humanized RadA,[1c] we set an initial hit cutoff value of +1.0 °C. The results from the screen, run in duplicate, are shown in Table 1.

**Table 1:** Results of initial DSF screening of the Maybridge Ro3 500-fragment library against *Pf*MTIPΔ60

	Set A	Set B	Both
**+ve** (Δ*T*_m_>1.0 °C)	134	126	112
**no change** (1.0 °C≥Δ*T*_m_≥−1.0 °C)	295	307	241
**−ve** (Δ*T*_m_<−1.0 °C)	18	22	13
**ambiguous** melting curve	53	45	44

The hit rate using this cutoff is remarkably high (26 %). It has been previously recognized that entropy-driven hydrophobic interactions generally result in a large Δ*T*_m_,[17] and this may be driven in the present case by exposed hydrophobic patches in the *Pf*MTIPΔ60 CTD. In view of the primary data, it was decided to increase the threshold to +2.0 °C in defining an initial hit. Furthermore, the hits were retested using DSF to improve confidence in the measured values. From 500 compounds, 40 were selected for retesting.

**Figure 2:**
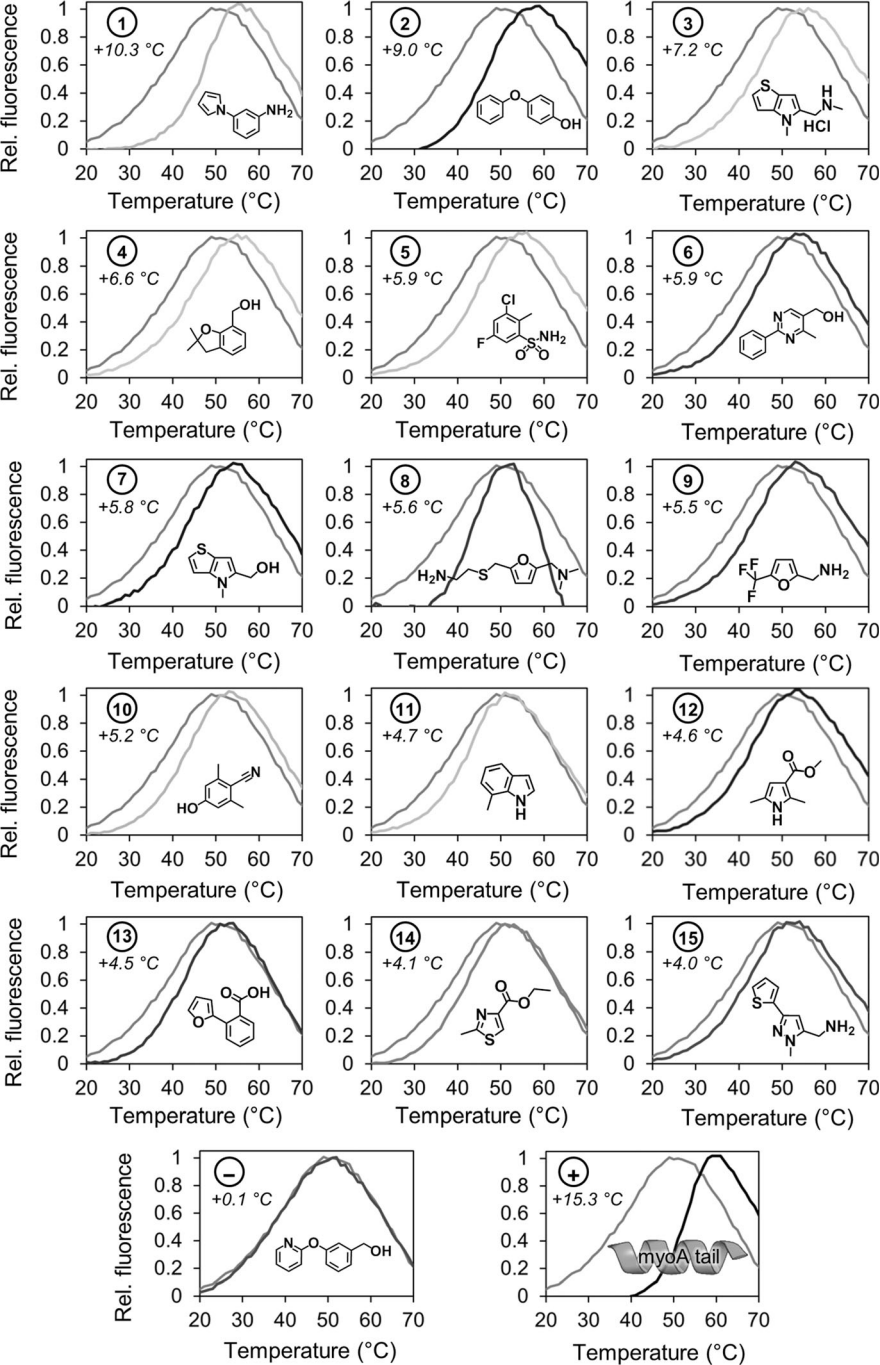
Normalized melting curves for *Pf*MTIPΔ60 (5 μm) in the presence of 1 % DMSO (grey) and the top 15 fragment hits (2 mm) and controls.

Of these 40 fragments, 15 were discounted upon retesting as they displayed a Δ*T*_m_<2.0 °C and/or inconsistent results (standard deviation >1.0 °C, *n*=4), ten gave Δ*T*_m_ between 2.0 and 4.0 °C, and 15 gave Δ*T*_m_>4.0 °C consistently. The latter 15 fragments (for which DSF data are shown in Figure 2) were selected for further testing. Substituted aniline **1** and 4-phenoxyphenol **2** caused the largest thermal shifts of the library, with remarkable Δ*T*_m_ values of +10.3 °C and +9.0 °C—just 5–6 °C lower than the positive control *Pf*myoA tail peptide despite the fragments being an order of magnitude smaller in molecular weight. Disubstituted furan **8** not only shifted the unfolding temperature of the protein, but also caused a marked sharpening of the melting curve, suggesting a significant change in the dynamics of MTIP unfolding. Of the top 25 fragments, seven contained one amine predicted to bear a positive charge in the assay buffer (pH 7.5) with a further two containing a second amine with p*K*_a_>7.5. In contrast, only one fragment hit is predicted to bear negative charge, with the remaining 15 hits neutral at this pH. *Pf*MTIPΔ60 has a predicted isoelectric point of 4.4, and the predominance of negatively charged residues in the sequence suggests an over-representation of amines may be expected. Otherwise, it is difficult to pick out clear trends for the hit compounds using the relatively crude measure of Δ*T*_m_.

### DSF titrations

**Figure 3:**
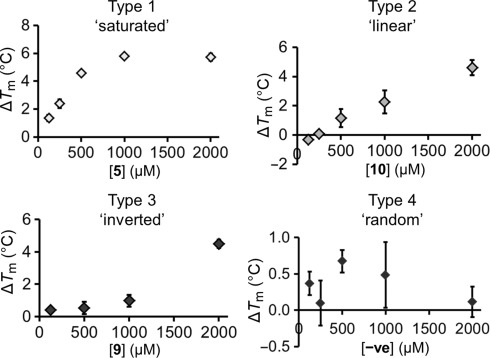
Examples of four different types of behavior in DSF titration experiments. Δ*T*_m_ values shown are the mean value of triplicate experiments, with error bars illustrating ± one standard deviation. For each fragment concentration, Δ*T*_m_ was referenced to the *T*_m_ value of a control in which the protein was incubated with the corresponding concentration of DMSO.

It was of interest to investigate whether the stabilization of *Pf*MTIPΔ60 observed in the initial DSF screen was dependent on the fragment concentration. Therefore, “titration” experiments were performed, in which fragment concentrations were varied from 125 to 2000 μm. Interestingly, several types of behavior were observed (Figure 3). For some fragment hits, the stabilizing effect was saturable, that is, Δ*T*_m_ increased with fragment concentration and reached a maximal value (‘type 1’). Several other hits showed a linear variation (‘type 2’) and some showed a marked jump to a large Δ*T*_m_ at high concentrations, but were barely or not at all stabilizing at lower concentrations (‘type 3’). Data for the 15 fragment hits are summarized in Table 2.

**Table 2:** Summary of DSF and NMR data for MTIP–fragment complexes

	Mean Δ*T*_m_ [°C]	DSF titration “type”	NMR sample pH	Amide peaks shifted *MTIP–myoA hotspots*^[a]^	Comments
DMSO ctrl	–	–	7.19	–	–
**−ve**	+0.1	4 (random)	7.02	D76, I133, A149	–
**1**	+10.3	1 (saturated)	7.05	D76, I133, A149, *D173*	nonspecific
**2**	+9.0	2 (linear)	7.04	I133, *N140*, V141, *G172*, *D173*, E190, I193	small CSPs
**3**	+7.2	2 (linear)	7.09	L68, E69, D73, E74, S75, D76, *S107*, *D173*	*validated*
**4**	+6.6	1 (saturated)	7.07	D76, A149, *D173*	nonspecific
**5**	+5.9	1 (saturated)	7.16	A180, C199, D201, *I202*	small CSPs
**6**	+5.9	3 (inverted)	7.13	D76, C134	nonspecific
**7**	+5.8	2 (linear)	7.08	D76, I133, A149	nonspecific
**8**	+5.6	1 (saturated)	7.09	D76, V135, *N140*, A149, H150, *T160*, *L168*, *W171*, *G172*, *D173*, *A174*, *T176*, D194, C199, *I202*	*validated*
**9**	+5.5	3 (inverted)	7.11	D76	nonspecific
**10**	+5.2	2 (linear)	7.14	C134, V141, A180	small CSPs
**11**	+4.7	1 (saturated)	7.12	D76, I133, A149, *D173*	nonspecific
**12**	+4.6	3 (inverted)	7.14	I133, A149, G157	nonspecific
**13**	+4.5	2 (linear)	6.94	D73, E74, D76, I133, C134, A149, H150, F151, *D173*	nonspecific
**14**	+4.1	3 (inverted)	7.10	I133	nonspecific
**15**	+4.0	3 (inverted)	7.11	I133	nonspecific

[a] Hotspots according to CSPs upon interaction with the myoA tail, illustrated in Figure 1 B.[14]

The effect of ligand concentration on protein stabilization and its relationship with binding affinity is complex and has been studied theoretically and experimentally.[18] It has been concluded that, in general, for 1:1 protein–ligand binding and a simple two-state approximation of the unfolding transition, Δ*T*_m_ should correlate reasonably with both affinity and ligand concentration. Furthermore, while the change in *T*_m_ may increase sharply as the protein is saturated by the ligand (particularly for tight binding) the increase of protein *T*_m_ with ligand concentration may continue after the protein (in its native state) is fully saturated.[18a] However, limits on ligand solubility or increased binding of the ligand to the denatured state may result in a saturation point, that is, type 1 behavior. Indeed, stabilization of the denatured state has been noted as a possible source of false negatives in DSF screens, as weak binding to the native state may be masked.[19] Another conclusion—working under the same two-state unfolding regime—is that no significant increase in Δ*T*_m_ should be observed for a ligand at a concentration well below its *K*_D_, which may lead to type 3 behavior for weaker-binding fragments. The highest affinity hits should, in principle, give the largest Δ*T*_m_ at a given concentration, as is commonly assumed, but may also be ranked according to titration behavior, with type 1>2>3.

There are several caveats in relation to these arguments, particularly for the target we are addressing. Firstly, the “foldedness” of *Pf*MTIPΔ60 at room temperature is complex because the protein is highly flexible, and the unfolding transition is shallow, suggesting that the protein samples a number of conformations.[14] As a result, the existing equations for modeling and predicting the effects of ligand binding to globular proteins in the thermal shift assays may be insufficient for such a target, and Δ*T*_m_ may correlate more poorly with affinity, particularly between different classes of molecules. The latter also appears to be the case for the diverse targets currently under investigation in our laboratories (data not shown). For instance, in the present case, it is improbable that fragment **1** has an affinity approaching that of the positive control *Pf*myoA tail peptide (*K*_D_=351 nm by ITC)[14] despite similar Δ*T*_m_ values (+10.1 and +15.3 °C, respectively). In the case of myoA peptide binding, a sharpening of the melting curve is associated with a major change in the structure and dynamics of the protein, that is, the unfolding energy landscapes of the two protein–ligand complexes are likely to be qualitatively different. Furthermore, the stoichiometry and reversibility of the MTIP unfolding transition are unknown, as is the character of the unfolded state of the protein. These concerns underline the particular importance of the validation stage of the screen, which is described in the next section.

### Validation of fragment binding by protein-detected NMR

Given the small size, domain structure and multifarious dynamics of isolated *Pf*MTIPΔ60, protein-detected experiments were deemed more appropriate than ligand-detected NMR experiments in the validation of initial fragment hits.[20] Binding of the 15 hit fragments to *Pf*MTIPΔ60 was monitored by single-point ^1^H,^15^N-HSQC experiments, in which the spectra of 50 μm ^15^N-labeled *Pf*MTIPΔ60 were recorded in the presence of 50 molar equivalents (2.5 mm) of the various fragments. These were compared with the spectrum of the protein in the presence of 1.25 % DMSO, to ensure that CSPs observed were not due to the presence of the solvent used to solubilize the fragment stocks. Despite slight changes in chemical shift for the protein in isolation in the presence of DMSO, these were mostly systematic and very minor such that the published peak assignments[14] could be adjusted accordingly and used with confidence. A negative control fragment was also analyzed in the same way. The pH of each of the samples (15 fragments and controls) was measured before the spectra were acquired, as small variations in pH may affect chemical shifts. CSPs were noted if the backbone amide resonance was shifted beyond its linewidth in either the ^1^H or ^15^N dimension (Table 2).

Overall, the CSPs observed in most of the protein–fragment HSQCs were qualitatively very small, which was surprising given the large values of Δ*T*_m_ obtained, and suggested either very weak interactions or no interactions with the fragments under the experimental conditions (Supporting Information Figure S1). Several residues appeared to be affected in nearly every sample; indeed, small peak shifts were observed in the negative control sample (Δ*T*_m_=+0.1 °C) for Asp 76, Ile 133, and Ala 149, which are presumed not to be caused by a direct interaction with the fragment. Chemical shifts are highly sensitive to pH in general—and residues lying close to the histidine residues in *Pf*MTIPΔ60 (His 136, His 150 and the N-terminal His tag) may be particularly affected due to the p*K*_a_ of the imidazole side chain; changes in the position of the equilibrium between protonated and unprotonated states will affect the local chemical environment and thus the chemical shifts of nearby nuclei. Two of the negative control peak shifts (Ile 133 and Ala 149) are thus likely due to pH, as the sample pH was 7.02, relative to 7.19 for the DMSO control. It is unclear why Asp 76, the amide chemical shift of which is affected in nearly every sample, should be likewise perturbed. Nonetheless, CSPs observed for these three amides were deemed to be nonspecific. The other indicator used to classify CSPs arising from a specific interaction was whether the affected amides were from a contiguous stretch in the primary MTIP sequence, although it should be noted that the lack of a structure for free *Pf*MTIPΔ60 prevents mapping of shifts in Table 2 onto a bona fide structural model of the protein.

Taking the above points together, several fragments were deemed to have insignificant or nonspecific interactions with MTIP on the basis of the NMR experiments. Notably, none of the fragments exhibiting type 3 DSF titration behavior were validated by NMR data. However, several fragments exhibiting type 1 or type 2 behavior were also not validated. It remains unclear why many DSF fragment hits fail to interact specifically with MTIP in the NMR experiment, but still appear to stabilize the protein significantly, and in a concentration-dependent manner. It is plausible that the fragments could stabilize the protein by binding to a conformation of MTIP that is not present (or is present as a minor, invisible conformation) in the NMR tube at 303 K.

**Figure 4:**
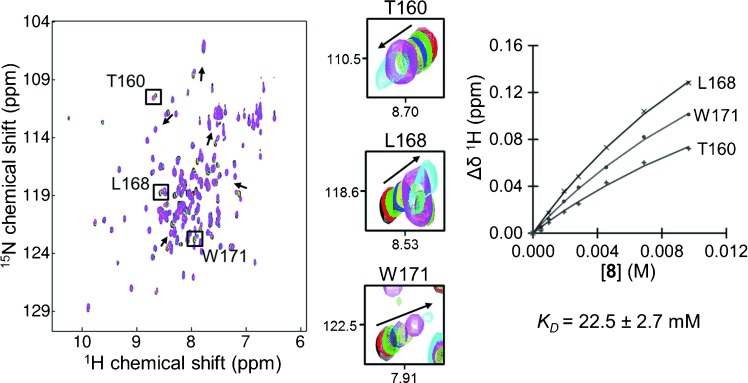
Measuring the binding affinity of hit 8 using protein-detected NMR. ^1^H,^15^N-HSQC spectra were recorded with 100 μm ^15^N-labeled *Pf*MTIPΔ60 before (black) and after addition of 8 at concentrations of approximately 5 (red), 10 (green), 20 (blue), 30 (yellow), 50 (pink), 80 (cyan) and 120 molar equivalents of the protein. Perturbations of several resonances were used to derive binding curves; three examples are highlighted. The *K*_D_ shown is the mean of the “per-residue” *K*_D_ values, quoted ± standard deviation (*n*=8).

Of the remaining fragments, by far the largest CSPs in MTIP were caused by hit **8**, a compound which caused not only a positive Δ*T*_m_ by DSF (+5.6 °C), but also a notable sharpening of the melting transition, and hit **3**. The interaction of **8** was further confirmed by running an NMR titration experiment, enabling measurement of its binding affinity (Figure 4). The CSPs of **8** localized to the MTIP CTD including several contiguous MTIP–myoA hotspots, for example, Leu 168, Trp 171, Gly 172, Asp 173, Ala 174, and Thr 176 that form the loop connecting helices α6 and α7, involved in the key intramolecular clamp with Ser 108 as described above. To define whether the binding mode was competitive with the myoA tail, another NMR experiment was carried out in which aliquots of **8** were added to a solution containing ^15^N-labeled *Pf*MTIPΔ60 bound to *Pf*myoA(799–818). No CSPs were observed in the presence of a large molar excess of the fragment, supporting the notion that **8** binds weakly to a part of MTIP that is engaged in stronger interactions with the myoA tail. When the same experiment was performed with **3**, which was shown in the initial NMR experiments with free MTIP to cause CSPs in several residues of the NTD distal to the myoA binding groove, clear CSPs were observed, suggesting that the interaction was not competitive with the myoA tail (Supporting Information Figure S2).

### Design of peptide–fragment chimeras

Although the affinity of **8** for *Pf*MTIPΔ60 was in the millimolar range according to NMR titration data, the clear interaction with the myoA binding hotspot loop connecting helices α6 and α7 was encouraging. With the aim of obtaining information on the mode of fragment binding, we synthesized a small library of derivatives based on **8** and assayed binding to MTIP by DSF (Table 3).

**Table 3:** DSF analysis to establish preliminary structure–activity relationships using derivatives of fragment hit 8

Compd	Structure	Δ*T*_m_ [°C]^[a]^
**8**	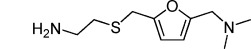	+5.6±0.7
**8 a**	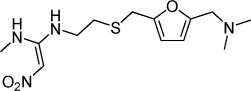	+1.2±0.6
**8 b**	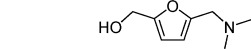	−1.4±0.7
**8 c**	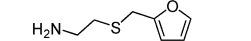	+3.0±1.3
**8 d**	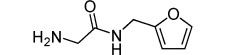	−5.7±0.9

[a] Values are quoted as the mean ±SD (*n*=3).

Compound **8** is a precursor to histamine receptor antagonist ranitidine (**8 a**). The latter gave a lower Δ*T*_m_ (+1.2 °C) than **8** (+5.6 °C), suggesting that the primary free amine is important to the interaction with MTIP. Replacement of this side of the molecule by an alcohol (**8 b**) severely decreased binding and actually led to destabilization (−1.4 °C) of the protein. However, removal of the dimethylamine moiety on the other side was better tolerated (**8 c**, Δ*T*_m_=+3.0 °C). Replacement of the thioether linker by an amide linker (**8 d**) was strongly destabilizing. Taken together, these results suggest that the primary amine linked to the furan via a thioether represent key features of **8**.

**Figure 5:**
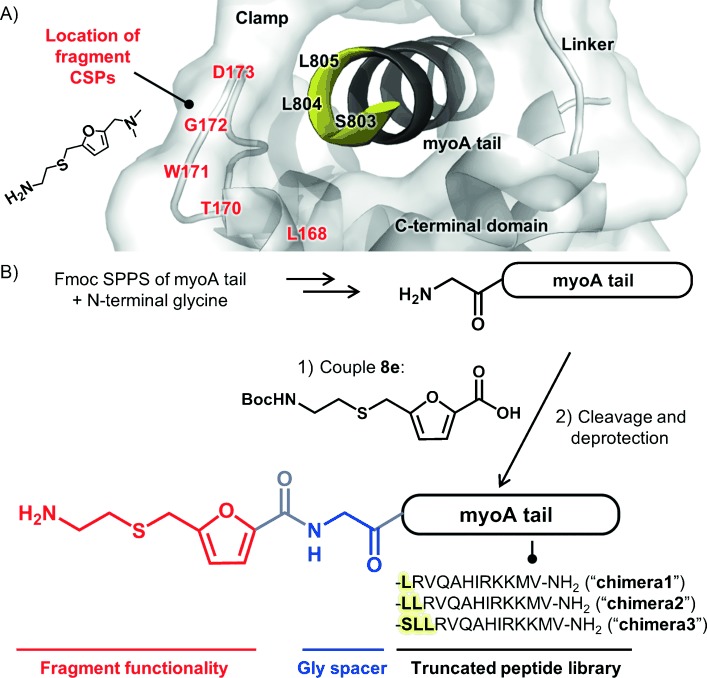
A) Design of peptide–fragment chimeras based on analysis of CSPs caused by fragment hit 8 (annotated red) and the crystal structure of the wild-type MTIP–myoA tail complex (PDB code: 4AOM). B) Scheme showing the synthesis and generalized structure of peptide–fragment chimera molecules.

We wished to examine the binding mode of **8** in more detail than afforded by the NMR validation experiments, which only gave an approximate binding site via backbone amide CSPs. However, previous studies strongly suggest that to crystallize *Pf*MTIPΔ60, a myoA tail peptide is required in order to fold the protein into a stable tertiary structure. In line with these observations, and despite the large stabilization induced by **8** according to DSF, saturation of free *Pf*MTIPΔ60 with fragment **8** did not promote crystal growth under any conditions tested (data not shown). However, given that one end of the molecule was not necessary for interaction with MTIP, it was hypothesized that attachment of the key moieties of **8** to a myoA tail peptide would enable an X-ray structure of the bound fragment to be obtained. Because the N-terminal part of *Pf*myoA(799–818) binds to the same site as the fragment, we systematically truncated the myoA tail at this end to create a library of chimeric molecules (Figure 5 A).

Synthesis of the chimeras (**chimera1**–**3**) was achieved via Fmoc-based solid-phase peptide synthesis, with a glycine spacer attached to the N terminus of each myoA sequence to permit flexibility in the orientation of the fragment. Final coupling of the key functionality of **8** using Boc-protected furan-2-carboxylic acid **8 e**, followed by TFA-mediated cleavage and deprotection, yielded the desired products (Figure 5 B). Three different myoA tail sequences were prepared (terminating at Leu 805, Leu 804, and Ser 803); by analysis of residue-specific CSPs caused by the fragment and the crystal structure of the wild-type complex with *Pf*myoA(799–818), it was rationalized that these positions should provide enough space for the fragment to adopt a binding conformation. Controls (**control1**–**3**) were also prepared in which the peptides were capped with acetyl groups.

### Structures of peptide–fragment chimeras bound to MTIP

Co-crystallization and thermal stability experiments were conducted for the three protein chimera complexes. Single crystals appeared under a number of conditions in each case within 48 h. Complete datasets were collected for the complexes of *Pf*MTIPΔ60 with **chimera2** and **chimera3**; the crystals containing **chimera1** diffracted at best to low (>3.5 Å) resolution and displayed unusual spot profiles; the dataset could not be further processed, and the structure remains unsolved. This may suggest a drastic change in the protein structure and/or crystal packing in the presence of this (shortest) chimera. Although the crystals of the **chimera3** and **chimera2** complexes appeared morphologically similar, the **chimera3** complex crystallized with *P*2_1_2_1_2_1_ symmetry (as for the wild-type complex) and diffracted to 1.98 Å resolution (Supporting Information Table S1), whereas the **chimera2** complex crystallized with *P*6_3_ symmetry and diffracted to 2.90 Å resolution (data not shown). The decreased number of crystal contacts in the latter led to a very high (∼70 %) solvent content.

**Figure 6:**
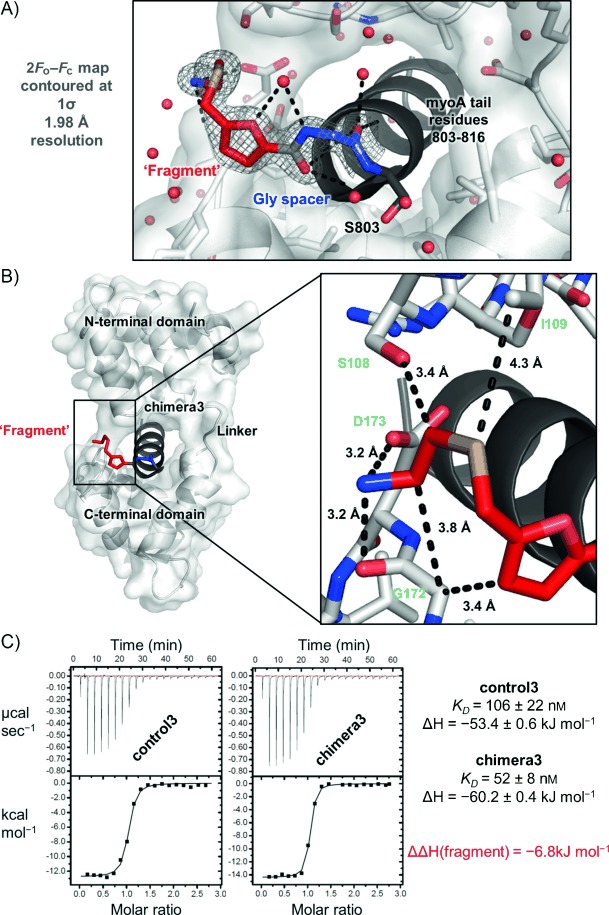
Structural and thermodynamic features of the interaction between MTIP and chimera3. A) Refined 2*F*_obs_−*F*_calc_ electron density map overlaid with the model of the non-natural functionality in peptide–fragment chimera3. B) Final crystal structure of the complex between *Pf*MTIPΔ60 (white surface and cartoon) and chimera3 (grey cartoon; glycine spacer shown as blue sticks, and fragment functionality shown as red sticks) and (inset) features of the complex, focusing on the interactions made by the fragment. C) ITC data for the interaction of *Pf*MTIPΔ60 with chimera3 and acetylated peptide control3. Data are quoted ± standard error (*n*=1).

The conformation of the protein in the X-ray structures is broadly the same as in that of the wild-type *Pf*MTIP–myoA tail complex.[11b, 14] In the **chimera2** complex, the fragment (**8**) moiety points toward solvent and does not appear to engage in interactions with the protein. Indeed, there is no electron density for the flexible thioether-containing chain that terminates in the important free primary amine (data not shown). By contrast, in **chimera3** the fragment-derived component makes a number of interactions with MTIP residues, and clear electron density is evident for all non-hydrogen atoms (Figure 6 A). Notably, the terminal free amine, identified as a key feature of **8**, forms a salt bridge with the side chain of Asp 173. This interaction does not perturb the hydrogen bonding clamp of the latter with Ser 108, which locks the stable protein conformation. The thioether moiety fits between Ile 109 and Gly 172, although the furan core does not make substantial interactions with MTIP (Figure 6 B). These results are supported by the observations that in the case of **chimera3**, the *T*_m_ of the complex (55.8 °C) is higher than that with the corresponding acetylated **control3** peptide (54.9 °C), presumably due to additional interactions observed in the X-ray structure, while the reverse is true for the **chimera2**/**control2** pair (53.4 and 54.5 °C respectively, Supporting Information Table S2). While the fragment moiety in **chimera1** also confers additional thermal stability (relative to **control1**) on the complex with MTIP, this could not be readily rationalized due to the absence of a structure. The *T*_m_ values of the latter complexes were much lower (47.8 and 45.4 °C, respectively), suggesting that this length of peptide sequence may be insufficient to induce the protein to adopt the stable, clamped fold observed in the other structures. To further validate the observed effect, ITC experiments were conducted with **control3** and **chimera3**. A two-fold increase in affinity was measured (106 and 52 nm, respectively), which appeared to be driven by an additional favorable enthalpic contribution from the fragment of 6.8 kJ mol^−1^ (Figure 6 C).

Taking these data in combination with the above NMR analysis, it is evident that **8** binds to the loop connecting helices α6 and α7. The extent to which the crystal structure of the **chimera3** complex reflects the orientation of free fragment **8** in this binding event remains an open question, as its orientation is governed to an unknown extent by its covalent attachment to the myoA tail peptide. Nevertheless, the high-resolution structure of **chimera3** opens some interesting possibilities for development of **8**. For example, rationally growing the fragment into the hotspot pockets occupied by side chains of nearby hydrophobic myoA residues Leu 804 and Val 807 may be an approach to generate a small library of peptidomimetic-type inhibitors. Alternatively, further optimization of interactions around the critical Asp 173/Ser 108 clamp may present an opportunity to modulate MTIP dynamics, and influence phosphorylation events in the vicinity of these residues that are thought to be important for control of the motor complex activity in vivo.[14]

## Conclusions

A 500-member fragment library was screened using DSF for interaction with MTIP, a critical determinant of malaria parasite actomyosin motor localization through its tight interaction with the tail domain of myosin A, and a potential target for novel antimalarials or tool compounds. The hit rate was remarkably high (26 %, for Δ*T*_m_>1 °C), reflecting the low thermal stability of the highly dynamic free MTIP construct and the lack of well-defined tertiary structure. This flexibility is manifest in a large loss of entropy upon binding its natural myoA partner, and in the shallow thermal unfolding transition suggesting a complex pathway that is probably not well-described by existing models of two-state unfolding.

As a result of the unusually high hit rate and large thermal shifts observed, the cutoff was increased to +4 °C in the DSF screen, leaving 15 fragments that were further tested. Surprisingly, despite the large (up to +10.3 °C) Δ*T*_m_ values and concentration-dependent stabilization given by these molecules, validation of binding by HSQC NMR experiments on MTIP suggested that the majority of fragments had negligible specific interactions with the protein. There are several possible explanations for this result. Firstly, it is possible that the hit fragments are false positives in the DSF experiment due to a destabilization of unfolded MTIP by an undetermined mechanism. Alternatively, the fragments bind to one or more minor conformation(s) of MTIP that are hardly populated at the experimental temperature (303 K) and are thus invisible in the NMR spectra. It is also possible that the affinity of the interactions with the “NMR-visible” protein conformer(s) is so low that chemical shift perturbations are undetectable. Nonetheless, at least one genuine “hit” fragment (**8**) was found that binds to the protein at the key loop connecting MTIP helices α6 and α7 in the CTD, a region that forms part of the intramolecular clamp with Ser 108 in the NTD and contains a number of myoA tail interaction hotspots.[14] Notably, this hit was the only molecule in the library that not only stabilized the protein to thermal unfolding significantly (Δ*T*_m_=+5.6 °C) but also caused a sharpening of the otherwise shallow melting transition. This suggests that the molecule binds to the protein in such a way as to quench a specific dynamic process and thus increase the cooperativity of unfolding. Another hit (**3**) bound at a site in the NTD that was independent of the interaction with the myoA tail. These are the first small molecules to have been identified and experimentally validated as MTIP binders.

High-resolution crystallographic data on fragment binding modes are often essential to rationally develop the affinity of hit molecules.[3] In the case of MTIP–myoA, this has proven to be problematic, as a crystal structure of free MTIP has not been resolved, and NMR data point to a highly flexible protein that samples a number of conformations, interconverting on different timescales. The data reported above highlight a significant challenge when the target is a protein–protein interaction in which the folding and stability of a protein is defined to a large extent by the presence of its natural partner protein or peptide. The screening and subsequent development of hits for such dynamic targets require innovative strategies; care must be taken in designing and interpreting the screening campaign and interpreting the biophysical data. For example, the high hit rate resulting from a DSF screen against a flexible protein like MTIP requires some consideration: rather than judging a hit solely from *shift* in melting temperature, it could be more productive to search for fragments that appear to change the cooperativity or slope of the transition, with the view that such molecules may be able to trap a dynamic region of the protein and thus promote particular (in this case, ideally non-myoA binding) conformations. To obtain necessary structural data for subsequent development, we first established the essential structure–activity relationship of the hit, and then synthesized novel peptide–fragment chimeras with flexible linkers both to “trap” a crystallizable conformation, and to probe the binding mode of the fragment functionality. This strategy allowed us to obtain a structure that provides an interesting starting point for the development of small molecules targeting key features of the MTIP clamp around the myoA motor.

## Experimental Section

**Differential scanning fluorimetry**: Samples (20 μL) were arrayed in 96-well real-time PCR plates (Eppendorf) and contained 5 μm *Pf*MTIPΔ60 expressed and purified as previously described[11d, 14] in buffer A (20 mm HEPES (pH 7.5), 50 mm NaCl, 1 mm TCEP), supplemented with 10× SYPRO Orange dye (Sigma–Aldrich). 500 fragments (Maybridge Ro3 library) were tested at 2 mm, and the melting curves were compared with negative controls containing 1 % DMSO; positive controls contained 200 μm peptide *Pf*myoA(803–818), synthesized as described previously.[11d] Where “titration” experiments were performed, the concentration of DMSO in controls was adjusted correspondingly. Experiments with peptide–fragment chimeras and controls used 5 μm *Pf*MTIPΔ60 and 200 μm peptide under otherwise identical conditions. Fluorescence of dye was monitored over 15–80 °C with a Mastercycler ep realplex real-time PCR instrument (Eppendorf). Melting curves were analyzed by using a customized MS Excel spreadsheet with a GraFit plug-in (Erithacus Software) to calculate Δ*T*_m_ for each molecule, by fitting to the Boltzmann equation.[17]

**Protein-detected NMR spectroscopy**: For single-point fragment validation experiments, ^1^H,^15^N-HSQC spectra were recorded in 3 mm NMR tubes (Norell) on 50 μm ^15^N-labeled *Pf*MTIPΔ60 expressed and purified as previously described[11d, 14] in buffer B (20 mm MOPS (pH 7.0), 50 mm NaCl, 1 mm TCEP), supplemented with 10 % D_2_O. Fragments were tested at 2.5 mm (added from a 50 mm stock in the same buffer) and the spectra were compared with a control containing DMSO (1.25 %). For the titration experiment with **8** and the binding experiments with the preformed MTIP–myoA complex, a 5 mm tube containing 100 μm ^15^N-labeled *Pf*MTIPΔ60 was used, and spectra were recorded before and after each addition of the ligand. All experiments were conducted at 303 K on a Bruker 600 MHz Avance III spectrometer equipped with a TCI cryoprobe. Spectra were processed in NMRPipe[21] and analyzed in NMRView.[22]

**Synthesis of fragment derivatives 8a–8d**: Ranitidine (**8 a**) was purchased from Sigma–Aldrich and was used without further purification. The synthesis of derivatives **8 b**, **8 c**, and **8 d** is described in the Supporting Information.

**Synthesis of peptide–fragment chimeras 1–3**: Synthesis of **chimera1**, **chimera2**, and **chimera3** and intermediate **8 e** is described in the Supporting Information.

**X-ray crystallography**: Complexes were formed between *Pf*MTIPΔ60 and the three peptide–fragment chimeras as follows: an aliquot of the protein in buffer A (300 μL, 100 μm) was mixed with the chimeric molecule (five molar equivalents in the case of **chimera2** and **chimera3**; ten molar equivalents in the case of **chimera1**), and the resulting complex was concentrated to 50 μL (600 μm) using a 3 kDa centrifugal concentrator (GE Healthcare) in buffer A. PEG/Ion sitting-drop crystallization screens (Hampton Research) were prepared in which the sitting drop contained 100 nL of protein and 100 nL of reservoir solution. The resulting crystals, which grew within 48 h at 293 K, were mounted in loops and cryoprotected using the reservoir solution supplemented with 30 % glycerol, flash frozen in liquid nitrogen and taken to Diamond Light Source synchrotron, Oxfordshire (UK) for data collection.

Data were collected at 100 K and with 1° oscillations, indexed in Mosflm and scaled in Scala.[23] Initial phasing by molecular replacement was carried out in Phaser[24] using the structure of wild-type *Pf*MTIP–myoA tail (PDB code: 4AOM) as the search model. The inspection of electron density maps and manual model building was done using Coot.[25] The non-natural fragment-derived functionality in **chimera3** was built using the PRODRG server.[26] Refinement was performed in REFMAC,[27] and model validation was done in Phenix.[28] The coordinates and structure factors for the complex between *Pf*MTIPΔ60 and **chimera3** were deposited in the RCSB Protein Data Bank under accession code 4R1E; statistics of data collection and refinement are shown in Supporting Information Table S1.

**Isothermal titration calorimetry**: Experiments were carried out at 303 K using a MicroCal iTC_200_ instrument (GE Healthcare). The cell contained ∼200 μL of 20 μm *Pf*MTIPΔ60 in buffer A; peptide ligands **control3** and **chimera3** were added in 2 μL injections from a 250 μm solution in buffer A. Data were fit to a single site binding event using Origin (OriginLab Corp).

## Supporting Information

As a service to our authors and readers, this journal provides supporting information supplied by the authors. Such materials are peer reviewed and may be re-organized for online delivery, but are not copy-edited or typeset. Technical support issues arising from supporting information (other than missing files) should be addressed to the authors.

miscellaneous_information
