# Supplementary material for: Targeting a Dynamic Protein–Protein Interaction: Fragment Screening against the Malaria Myosin A Motor Complex
Source: ChemMedChem. 2014 Nov 3;10(1):134–43. doi: 10.1002/cmdc.201402357 (PMC4506568; doi:10.1002/cmdc.201402357)
Supplement: Supplementary file 1 — miscellaneous_information [file cmdc0010-0134-sd1.pdf]

CHEMMEDCHEM
Chemistry & Drug Discovery

Supporting Information

© Copyright Wiley-VCH Verlag GmbH & Co. KGaA, 69451 Weinheim, 2014

Targeting a Dynamic Protein–Protein Interaction: Fragment Screening against the Malaria Myosin A Motor Complex

Christopher H. Douse,*[a, b, c] Nina Vrielink,[a] Zhang Wenlin,[a] Ernesto Cota,[b, c] and Edward W. Tate*[a, c]

cmdc_201402357_sm_miscellaneous_information.pdf

Supplementary Figures and Tables

**Protein**	*Pf*MTIPΔ60
**Ligand**	'chimera3'
Reservoir condition	200 mM $(NH_4)_2SO_4$<br>20% PEG 3,350
**Data collection**	
X-ray source	Diamond Light Source Beamline i04-1
Space group	$P2_12_12_1$
Unit cell dimensions	
a, b, c (Å)	36.71, 55.76, 75.86
$\alpha = \beta = \gamma = 90°$	
Resolution (Å)	44.93 - 1.98 (2.09 - 1.98)
Observations	33333
Unique reflections	11020
R_{merge}	0.041 (0.106)
$\langle I \rangle / \sigma(I)$	13.2 (6.9)
Completeness (%)	97.6 (97.9)
Redundancy	3.0 (3.2)
**Refinement**	
Protein molecules in a.u.	1
R_{work} / R_{free} (%)	20.4 / 22.9
No. atoms	
Protein	1070
Ligand	140
Water	66
Mean B-factor (Å^2)	18.4
R.m.s. deviations	
Bond lengths (Å)	0.008
Bond angles (°)	1.1
Ramachandran statistics	
% favoured	100.0
% allowed	0.0
% outliers	0.0
**PDB code**	4R1E

*Values in parentheses are for the highest-resolution shell.

Table S1 X-ray Data Collection and Refinement Statistics

R=	Molecule	mean T_m (°C) of complex
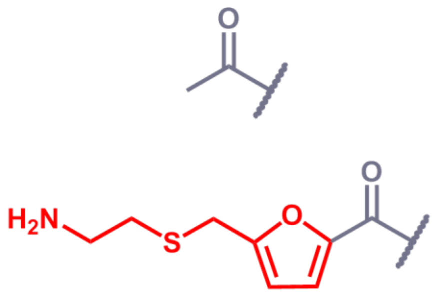	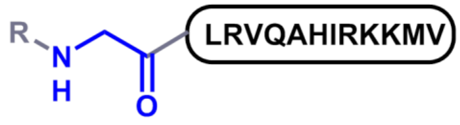	**control1**<br>45.4 ± 0.2<br>↓ ***+2.4°***<br>47.8 ± 0.2<br>**chimera1**
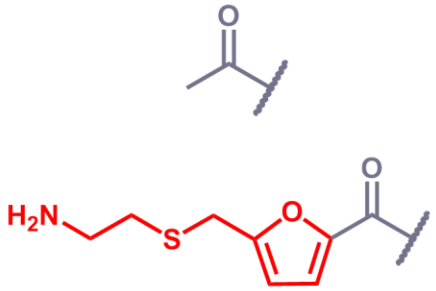	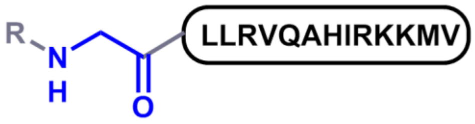	**control2**<br>54.5 ± 0.3<br>↓ ***-1.1°***<br>53.4 ± 0.5<br>**chimera2**
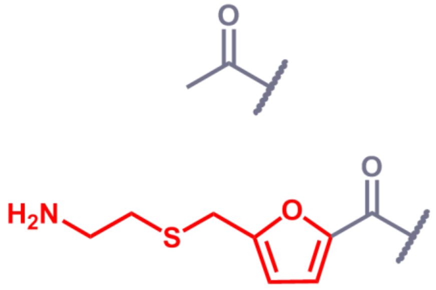	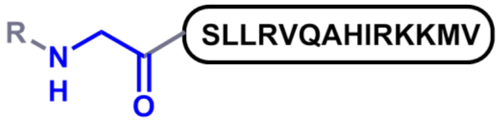	**control3**<br>54.9 ± 0.3<br>↓ ***+0.9°***<br>55.8 ± 0.2<br>**chimera3**

Table S2 DSF analysis of three lengths of peptide-fragment chimeras binding to MTIP. T_m values of complexes are quoted as mean ± standard deviation (n = 6), and the difference between the chimera and the acetylated control noted in each case.

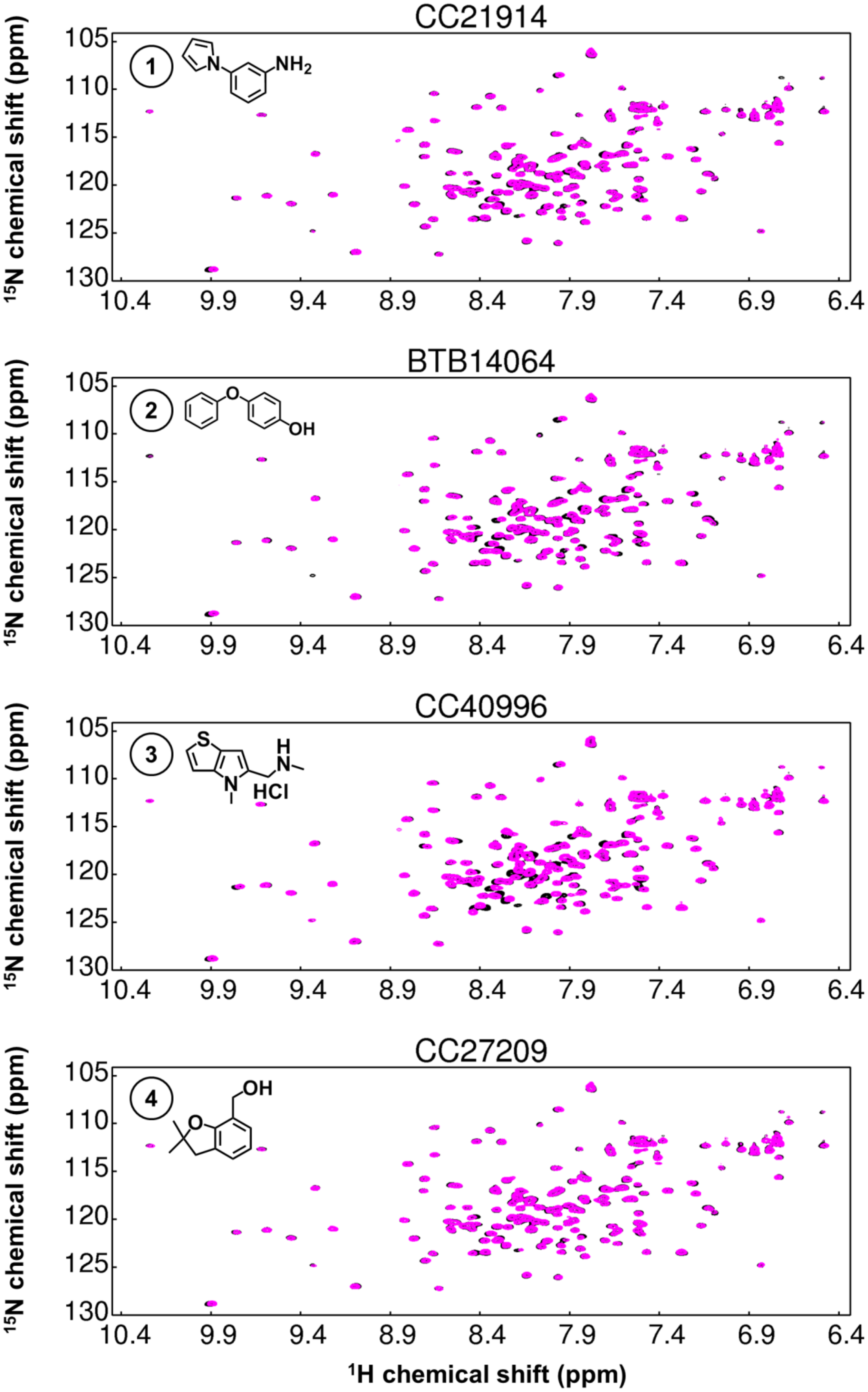

CC21914
1
NH2
N
BTB14064
2
O
OH
CC40996
3
S
H
N
N
HCl
CC27209
4
OH
O
105
110
115
120
125
130
10.4
9.9
9.4
8.9
8.4
7.9
7.4
6.9
6.4
15N chemical shift (ppm)
1H chemical shift (ppm)

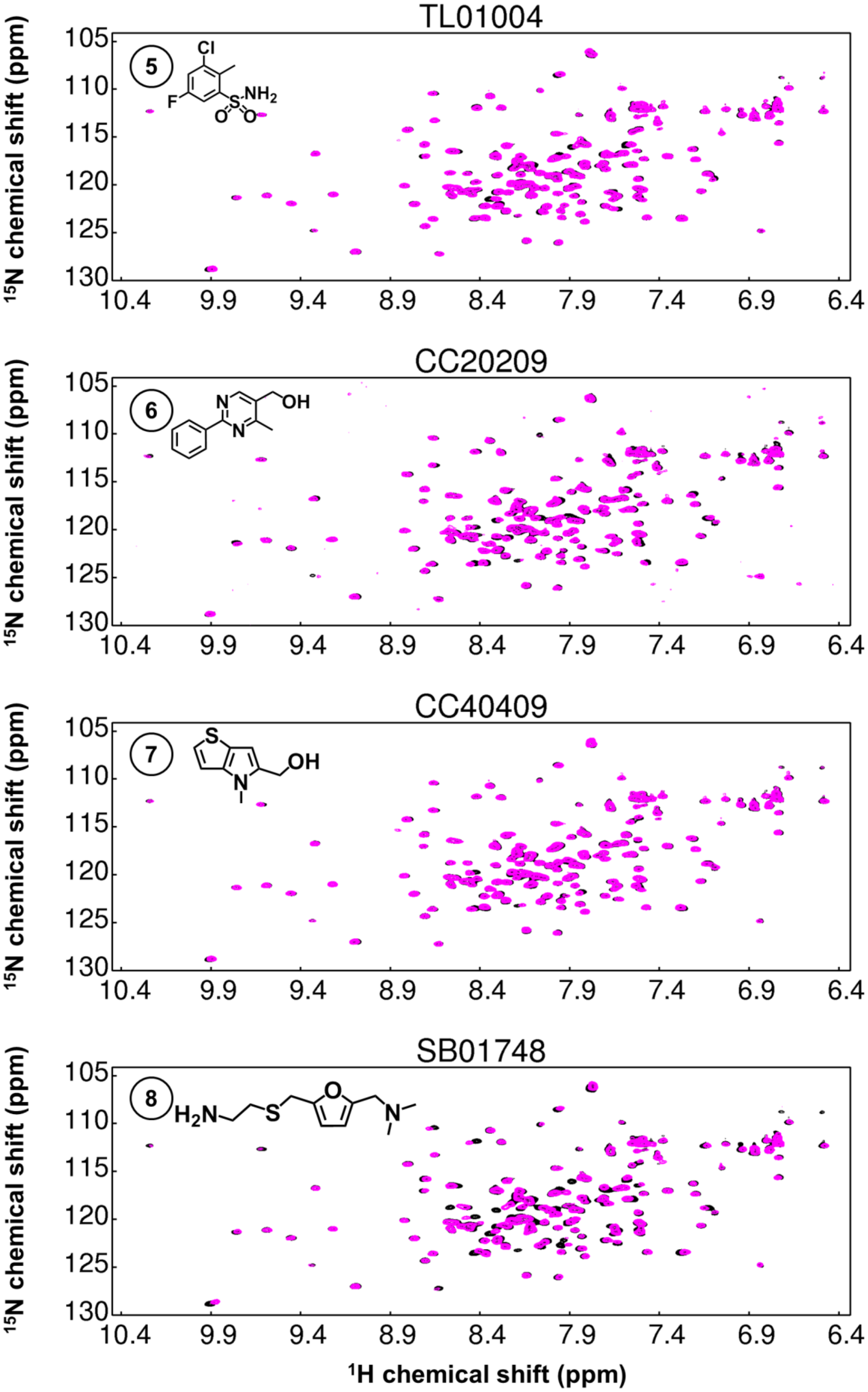
TL01004
5
Cl
F
NH2
S
O
O
CC20209
6
N
N
OH
CC40409
7
S
N
OH
SB01748
8
H2N
S
O
N
105
110
115
120
125
130
10.4
9.9
9.4
8.9
8.4
7.9
7.4
6.9
6.4
15N chemical shift (ppm)
1H chemical shift (ppm)

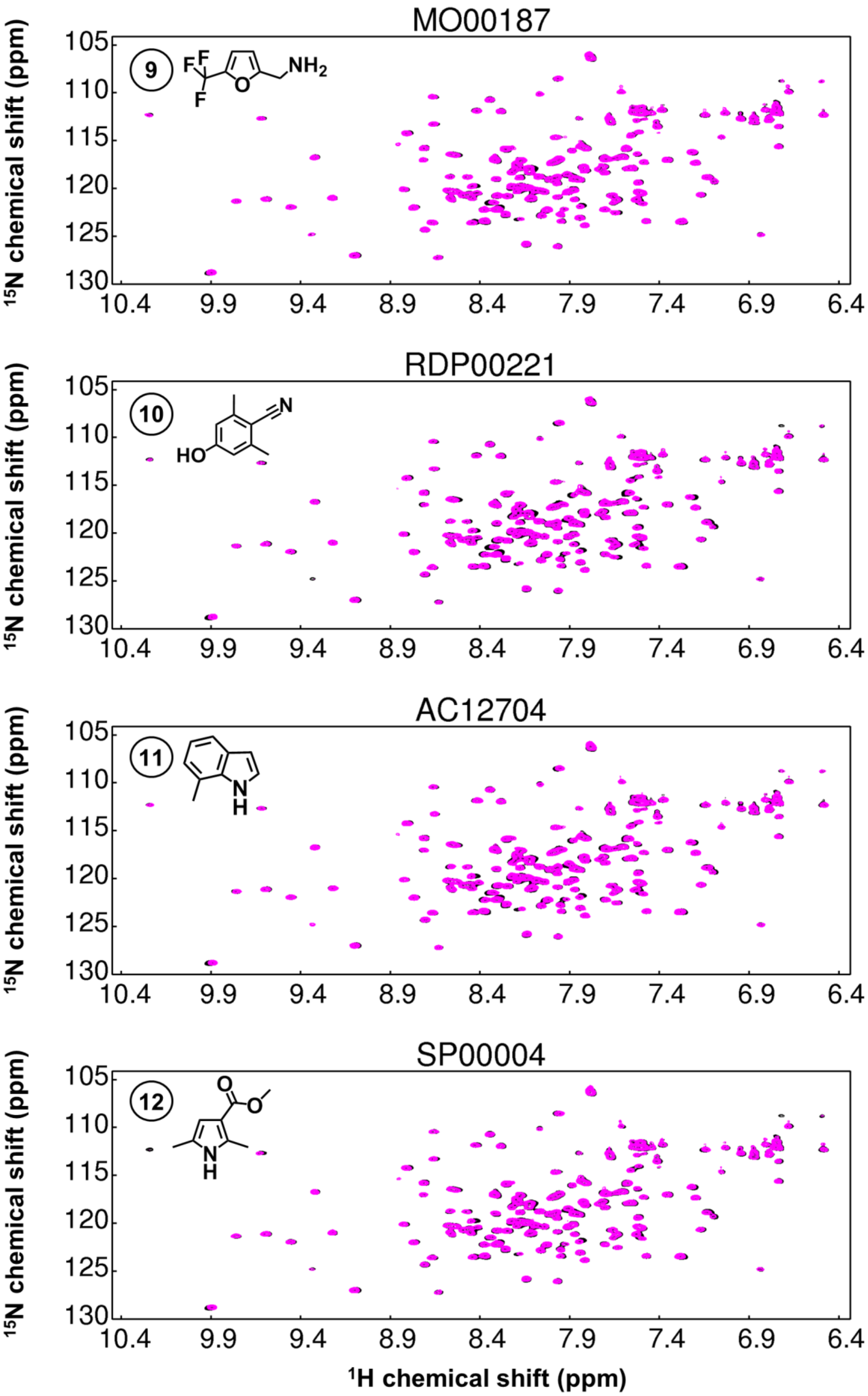

MO00187
9
RDP00221
10
AC12704
11
SP00004
12
15N chemical shift (ppm)
1H chemical shift (ppm)
105
110
115
120
125
130
10.4
9.9
9.4
8.9
8.4
7.9
7.4
6.9
6.4

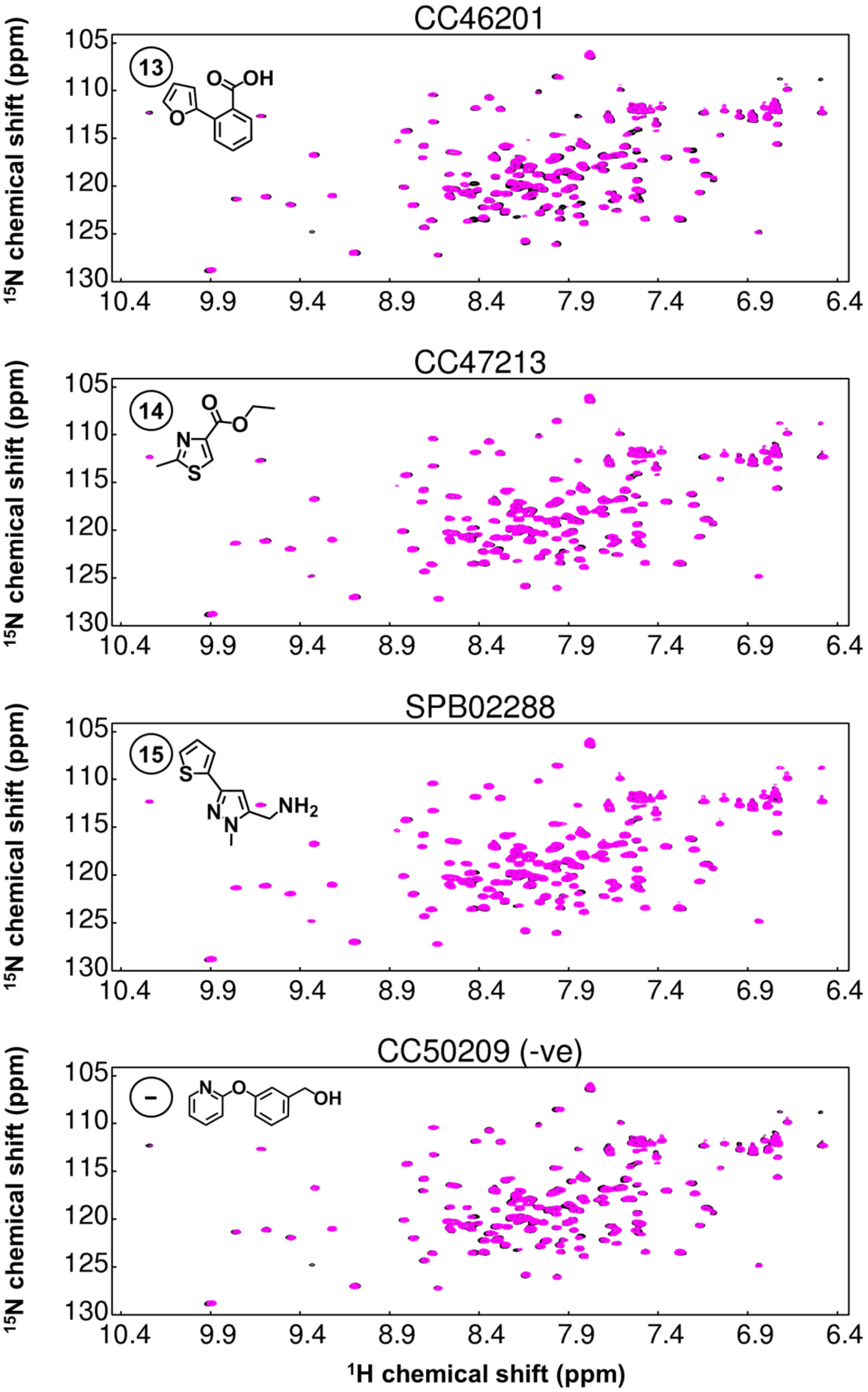

CC46201
13
O OH
O
CC47213
14
O
O
N
S
SPB02288
15
S
N
N
NH2
CC50209 (-ve)
–
N
O
OH
105
110
115
120
125
130
15N chemical shift (ppm)
10.4
9.9
9.4
8.9
8.4
7.9
7.4
6.9
6.4
1H chemical shift (ppm)

Figure S1 (previous four pages) Overlaid ^{1}H, ^{15}N-HSQC spectra of ^{15}N-labelled *Pf*MTIPΔ60 (50 μM) in the presence of 1.25% (v/v) DMSO (black) and 2.5 mM of various fragments (pink). The spectra are presented in order of ranking based on ΔT_m from the initial DSF screen, followed by the negative control fragment (Maybridge code CC50209).

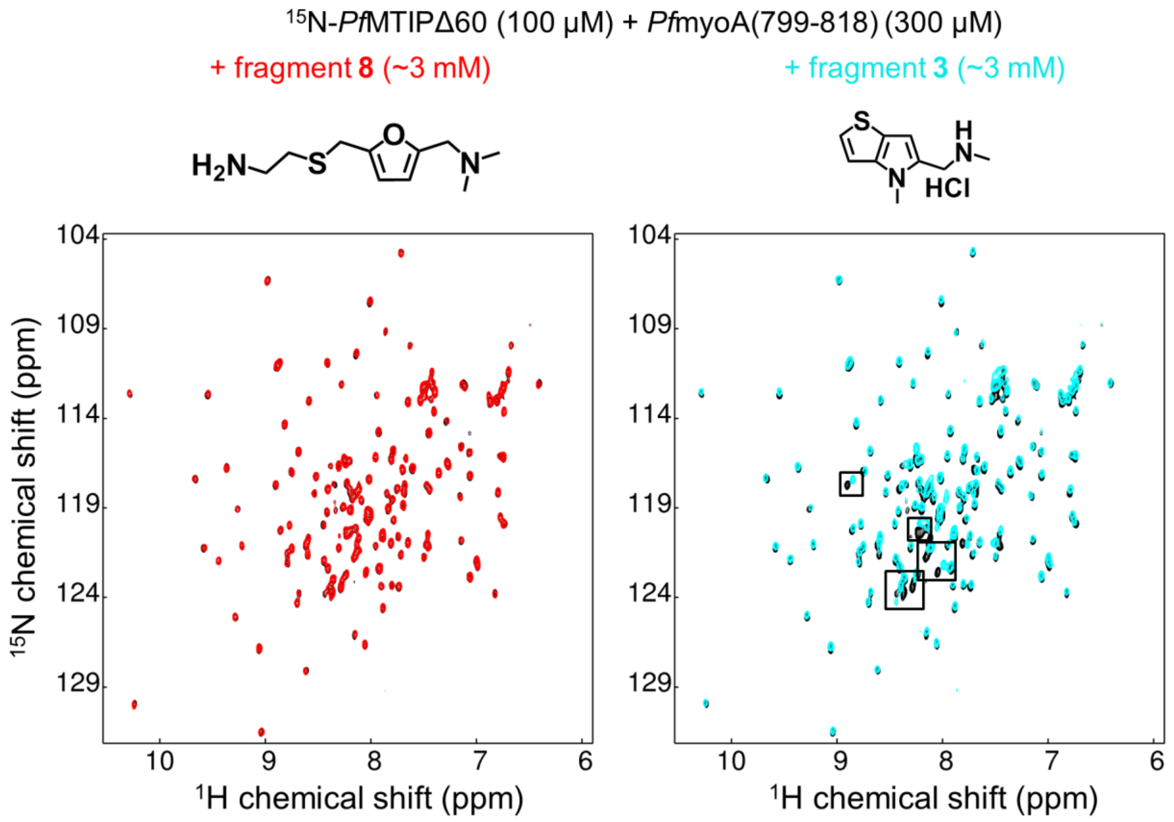

Figure S2 Fragment hit binding to a preformed MTIP-myoA complex. Overlaid ^{1}H, ^{15}N-HSQC spectra of 100 μM ^{15}N-labelled *Pf*MTIPΔ60 in the presence of 300 μM unlabelled *Pf*myoA(799-818) (black) with the addition of ~3 mM of fragment hits **8** (red, left) or **3** (cyan, right). Small chemical shift perturbations caused by non-competitive binding of **3** are highlighted in boxes.

Synthetic details

All chemicals and solvents were obtained from chemical suppliers (Sigma Aldrich, VWR International, Fisher Scientific) and used without further purification. Unless otherwise noted, all reaction mixtures were magnetically stirred in oven-dried glassware. External bath temperatures were used to record all reaction mixture temperatures. Water sensitive reactions were performed in dry solvents. Reactions that required an inert atmosphere were carried out under nitrogen gas (N_2). Ultrapure water was obtained from a MilliQ® Millipore water purification system. To remove water, compounds were lyophilized with an Alpha 2-4 LD Plus freeze-dryer (Christ).

NMR spectra of the small molecules were recorded in 5 mm tubes on a Bruker AM-400 spectrometer in deuterated solvents at room temperature. The spectra were referenced to the solvent residual peak. Chemical shifts are given as δ values in ppm (parts per million) and coupling constants for ^1H NMR are assigned in Hz, if possible. The following abbreviations are used to explain the multiplicities: s = singlet, d = doublet, t = triplet, q = quartet, m = multiplet, br = broad.

Analytical and semi-preparative LC-MS used various gradients of MeOH:H_2O over 18 min; the solvents were degassed and supplemented with 0.1% formic acid prior to use. The LC-MS platform consisted of a Waters RP-HPLC system (Waters 2767 autosampler for sample injection and collection; Waters 515 HPLC pump to deliver the mobile phase to the source; XBridge C_{18} columns with dimensions 4.6 mm x 100 mm for analytical runs and 19 mm x 100 mm for preparative runs) coupled to a Waters 3100 mass spectrometer (with ESI in positive and negative modes) and a Waters 2998 Photodiode Array (with detection between 200-600 nm).

Low resolution mass analysis and purification was performed using a Waters LC-MS platform consisting of a Waters RP-HPLC system (Waters 2767 autosampler for sample injection and collection; Waters 515 HPLC pump to deliver the mobile phase to the source; XBridge C_{18} columns with dimensions 4.6 mm x 100 mm for analytical runs and 19 mm x 100 mm for preparative runs) coupled to a Waters 3100 mass spectrometer (with ESI in positive and negative modes) and a Waters 2998 Photodiode Array (with detection between 200-600 nm). High resolution mass spectrometry (HRMS) was performed on an AUTOSPEC P673 spectrometer by electrospray ionization.

Solid-phase peptide synthesis was carried out on a ResPep SL automated multiple peptide synthesizer from Intavis. All Fmoc-protected amino acids, resins, coupling reagents and solvents were purchased from Merck Chemicals. Peptide mass spectrometric characterization was carried out using the Waters LC-MS system described above.

Synthesis of fragment derivatives 8b-8d

The routes to fragment derivatives **8b-8d** are summarized in Scheme S1.

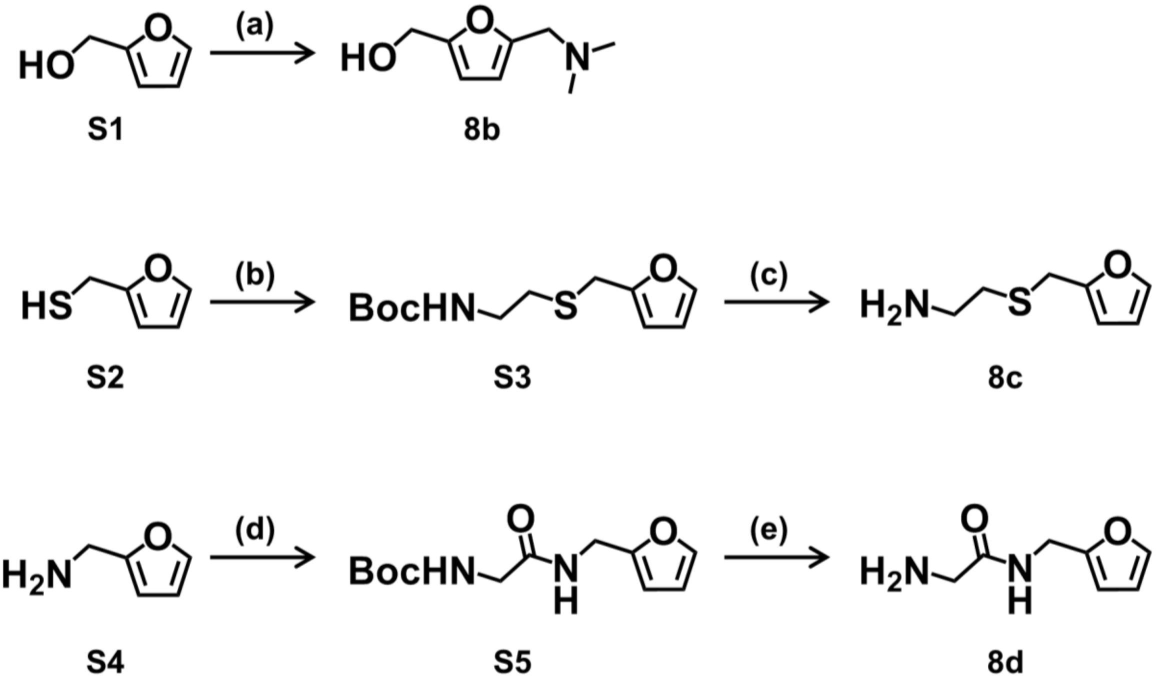

Scheme S1 Synthesis of **8b**, **8c** and **8d**. a) (bisdimethylamino)methane (1.1 eq.), AcOH, r.t., 18 h; b) *N*-Boc-bromoethylamine (1.1 eq.), K_2CO_3 (1.1 eq.), DMF .), r.t., 24 h; c) HCl (1 M in Et_2O), r.t., 2 h; d) *N*-Boc-glycine (1.0 eq.), DIPEA (1.1 eq.), PyBOP (1.1 eq.), CH_2Cl_2, r.t., 4 h; e) HCl (1 M in Et_2O), r.t., 12 h.

5-(Dimethylaminomethyl)furfuryl alcohol (8b)

To a stirred solution of (bisdimethylamino)methane (560 mg, 5.50 mmol, 1.1 eq.) in AcOH (1 mL) was added dropwise a solution of furan-2-ylmethanol (**S1**, 480 mg, 5.00 mmol, 1.0 eq.) in AcOH (5 mL) at 10 °C. The mixture was stirred at room temperature for 18 h and another portion of (bisdimethylamino)methane (50.0 mg, 0.480 mmol, 0.1 eq.) was added. After 20 h AcOH was removed *in vacuo*. Ice was added to the residue, followed by addition of aqueous NaOH (40% w/v) until the pH of the reaction mixture was greater than 7. The mixture was extracted with EtOAc (3 x 20 mL) and the combined organic layers were washed with saturated aqueous NaCl (30 mL) dried over Na_2SO_4 and concentrated *in vacuo* to afford 5-(Dimethylaminomethyl)furfuryl alcohol (**8b**, 760 g, 4.89 mmol, 89% yield) as a brown viscous oil.

^{1}H NMR (400 MHz, $CDCl_3$): δ=6.21 (1 H, d, $^3J_{HH}$ = 3.1 Hz, Fur-*H*), 6.14 (1 H, d, $^3J_{HH}$ = 3.1 Hz, Fur-*H*), 4.57 (2 H, s, Fur-CH_2-OH), 3.43 (2 H, s, Fur-CH_2-NMe_2), 2.25 (6 H, s, -N(CH_3)$_2$), 1.99 (1 H, br s, Fur-CH_2-O*H*) ppm.

The analytical data matched those previously described in the literature [P. A. Haywood, M. M. Smith, T. J. Cholerton, M. B. Evans *J. Chem. Soc., Perkin Trans. 1* **1987**, 951–954].

***N*-Boc-2-((furan-2-ylmethyl)thio)ethanamine (S3)**

2-Furylmethanethiol (**S2**, 100 mg, 0.876 mmol, 1.0 eq.) and *N*-Boc-bromoethylamine (216 mg, 0.964 mmol, 1.1 eq.) were dissolved in DMF (4 mL) and K_2CO_3 (133 mg, 0.964 mmol, 1.1 eq.) was added. The reaction was stirred for 24 h at room temperature; afterwards the organic layer was washed with saturated aqueous $NaHCO_3$ (2 x 10 mL) and dried over Na_2SO_4. The solvents were removed under reduced pressure and the residue was purified by flash column chromatography (hexanes:EtOAc = 9:1) to yield *N*-Boc-2-((furan-2-ylmethyl)thio)ethanamine (**S3**, 56.9 mg, 0.221 mmol, 19% yield).

$\mathbf{R}_f$ = 0.50 [hexanes:EtOAc = 9:1].

^{1}H NMR (400 MHz, $CDCl_3$): data for major rotamer reported. δ=7.33 (1 H, dd, $^4J_{HH}$ = 0.8, $^3J_{HH}$ = 1.8 Hz, Fur-*H*), 6.28 (1 H, dd, $^3J_{HH}$ = 1.9, 3.2 Hz, Fur-*H*), 6.17 (1 H, dd, $^4J_{HH}$ = 0.5, $^3J_{HH}$ = 3.2 Hz, Fur-*H*), 3.70 (2 H, s, Fur-C*H*$_2$), 3.24 (1 H, br t, $^3J_{HH}$ = 6.3 Hz, CH_2-N*H*Boc), 2.60 (2 H, t, $^3J_{HH}$ = 6.5 Hz, S-C*H*$_2$-CH_2), 1.41 (9 H, s, $^3J_{HH}$ = 5.0 Hz, -C(C*H*$_3$)$_3$), 0.85 (2 H, t, $^3J_{HH}$ = 6.9 Hz, C*H*$_2$-NHBoc) ppm.

HRMS (EI): calcd. for $C_{12}H_{19}NO_3S$ 258.1158 $[M + H]^+$
found: 257.9995 $[M + H]^+$.

2-((Furan-2-ylmethyl)thio)ethanamine (8c)

Boc-protected amine **S3** (59.6 mg, 0.221 mmol) was dissolved in Et_2O (2 mL). Upon addition of HCl (2 M in Et_2O, 2 mL), a white precipitate formed immediately. After stirring for 2 h at room temperature, the solvent was removed *in vacuo*. The crude product was dissolved in water (1 mL) and purified by preparative LC-MS to yield 2-((furan-2-ylmethyl)thio)ethanamine (**8c**, 2.1 mg, 13 µmol, 6% yield).

^{1}H NMR (400 MHz, MeOD): δ=7.42 (1 H, s, Fur-*H*), 6.33 (1 H, s, Fur-*H*), 6.26 (1 H, d, $^3J_{HH}$ = 2.8 Hz, Fur-*H*), 3.79 (2 H, s, Fur-C*H*$_2$-S-), 3.01 (2 H, t, $^3J_{HH}$ = 6.8 Hz, -S-C*H*$_2$), 2.74 (2 H, t, $^3J_{HH}$ = 6.8 Hz, C*H*$_2$-NH_2) ppm.

***tert*-Butyl(2-((furan-2-ylmethyl)amino)-2-oxoethyl)carbamate (S5)**

N-Boc glycine (1.00 g, 5.71 mmol, 1.0 eq.), DIPEA (811 mg, 6.28 mmol, 1.1 eq.) and PyBOP (3.27 g, 6.28 mmol, 1.1 eq.) were dissolved in CH_2Cl_2 (30 mL) and stirred for 20 min. Afterwards, furfurylmethanolamine (**S4**, 610 mg, 6.28 mmol, 1.1 eq.) was added and the reaction mixture was stirred for another 4 h. Water (20 mL) was added and the aqueous phase was extracted with EtOAc (3 x 20 mL). The organic layers were combined, washed

with saturated, aqueous NaCl (10 mL), dried over $MgSO_4$ and concentrated *in vacuo*. The crude product was purified by flash column chromatography (hexanes:EtOAc = 3:2) to yield *tert*-butyl(2-((furan-2-ylmethyl)amino)-2-oxoethyl)carbamate (**S5**, 658 mg, 0.453 mmol, 45% yield) as white crystals.

$\mathbf{R}_{\boldsymbol{f}}$ = 0.51 [hexanes:EtOAc = 3:2].

^{1}H NMR (400 MHz, $CDCl_3$): δ=7.33 (1 H, dd, $^4J_{HH}$ = 0.8, $^3J_{HH}$ = 1.8 Hz, Fur-*H*), 6.44 (1 H, br s, N*H*), 6.29 (1 H, dd, $^3J_{HH}$ = 1.9, 3.2 Hz, Fur-*H*), 6.21 (1 H, d, $^3J_{HH}$ = 2.8 Hz, Fur-*H*), 5.10 (1 H, br s, N*H*), 4.43 (2 H, d, $^3J_{HH}$ = 5.5 Hz, Fur-CH_2-NH), 3.79 (2 H, s, CH_2-NHBoc), 1.41 (9 H, s, C(CH_3)$_3$) ppm.

**HRMS** (EI):	calcd. for $C_{12}H_{18}N_2O_4$	277.1159 $[M + Na]^+$
	found:	277.1166 $[M + Na]^+$.

2-Amino-*N*-(furan-2-ylmethyl)acetamide (8d)

Boc-protected amine **S5** (200 mg, 0.787 mmol) was dissolved in Et_2O (2 mL) and upon addition of HCl (2 M in Et_2O, 2 mL).), a white precipitate was formed after stirring for 12 h at room temperature,, which was filtered and washed with cold Et_2O. The crude product was dissolved in water (1 mL) and purified by preparative LC-MS to yield 2-amino-*N*-(furan-2-ylmethyl)acetamide (**8d,** 78.1 mg, 0.507 mmol, 64% yield).

^{1}H NMR (400 MHz, MeOD): δ=7.43 (1 H, s, Fur-*H*), 6.35 (1 H, s, Fur-*H*), 6.30 (1 H, d, $^3J_{HH}$ = 2.3 Hz, Fur-*H*), 4.43 (2 H, s, Fur-CH_2-), 3.69 (2 H, s, H_2N-CH_2- or H_2N-CH_2-), 3.33 (2 H, s, H_2N-CH_2- or H_2N-CH_2-) ppm.

^{13}C NMR (101 MHz, MeOD): δ=166.96, 152.37, 143.57, 111.53, 108.55, 41.74, 37.30 ppm.

HRMS (EI):	calcd. for $C_7H_{10}N_2O_2$	155.0815 $[M + H]^+$
	found:	155.0812 $[M + H]^+$.

Synthesis of peptide-fragment chimeras and controls

Synthesis of fragment derivative 8e

The route to fragment derivative **8e** is summarized in Scheme S2.

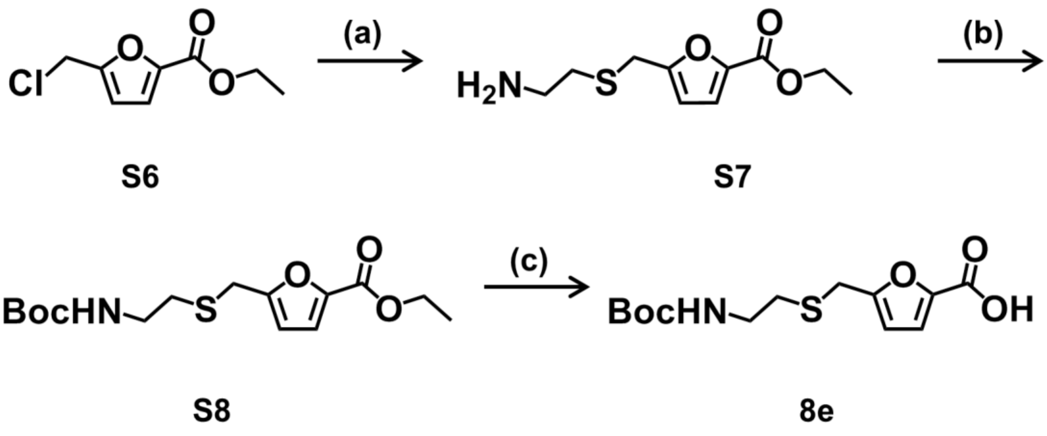

Scheme S2 Synthesis of **8e**. a) 2-Aminoethanethiol.HCl (1.1 eq.), K_2CO_3 (2.2 eq), DMF, r.t., 12 h; b) Boc_2O (1.0 eq.), NEt_3 (2.0 eq.), CH_2Cl_2, r.t.,12 h; c) NaOH (2.0 eq.), MeOH, 60 °C, 4 h.

Ethyl-5-(((2-aminoethyl)thio)methyl)furan-2-carboxylate (S7)

2-Aminoethanethiol.HCl (662 mg, 5.83 mmol, 1.1 eq.) was dissolved in dry DMF (4 mL) and K_2CO_3 (1.63 g, 11.7 mmol, 2.2 eq.) was added at 0 °C. After 20 min, ethyl-5-(chloromethyl)-2-furoate (**S6**, 1.00 g, 5.30 mmol, 1.0 eq.) was added and the reaction was allowed to warm to room temperature. After 12 h, the solvent was removed under reduced pressure and the residue was dissolved in EtOAc (15 mL). The organic layer was washed with water (15 mL) and the aqueous layer was extracted with EtOAc (2 x 20 mL); afterwards the organic layers were combined and the solvent was removed *in vacuo*. This procedure was repeated three times. After the last extraction, the combined organic layers were dried over Na_2SO_4 and concentrated *in vacuo* to afford ethyl-5-(((2-aminoethyl)thio)methyl)furan-2-carboxylate (**S7**, 1.14 g, 4.99 mmol, 94% yield) as a colourless oil.

$\mathbf{R_f}$ = 0.32 [CH_2Cl_2:MeOH = 5:1 + 1% NEt_3].

^{1}H NMR (400 MHz, MeOD): δ=7.16 (1 H, d, $^3J_{HH}$ = 3.3 Hz, Fur-*H*), 6.47 (1 H, d, $^3J_{HH}$ = 3.0 Hz, Fur-*H*), 4.32 (2 H, q, $^3J_{HH}$ = 7.1 Hz, -CH_2CH$_3$), 3.81 (2 H, s, Fur-CH_2-S), 2.81 (2 H, t, $^3J_{HH}$ = 6.5 Hz, S-CH_2), 2.67 (2 H, t, $^3J_{HH}$ = 6.5 Hz, CH_2-NH$_2$), 1.35 (3 H, t, $^3J_{HH}$ = 7.1 Hz, -CH$_2$CH_3) ppm.

^{13}C NMR (101 MHz, MeOD): δ=160.31, 158.86, 145.33, 120.33, 110.92, 62.33, 41.39, 35.40, 28.55, 14.76 ppm.

**HRMS** (EI):	calcd. for $C_{10}H_{15}NO_3S$	230.0845 [M + H]$^+$
	found:	230.0850 [M + H]$^+$.

Ethyl-5-(((*N*-Boc-2-aminoethyl)thio)methyl)furan-2-carboxylate (S8)

Ethyl-5-(((2-aminoethyl)thio)methyl)furan-2-carboxylate (**S7**, 1.00 g, 4.88 mmol, 1.0 eq.) and Boc_2O (1.05 g, 4.88 mmol, 1.0 eq.) were dissolved in dry CH_2Cl_2 (17 mL) and cooled to 0 °C. NEt_3 (1.40 mL, 9.76 mmol, 2.0 eq.) in dry CH_2Cl_2 (3 mL) was added . After 10 min the reaction was allowed to warm to room temperature and stirred for 12 h. The reaction was stopped by addition of water (10 mL) and the aqueous phase was extracted with EtOAc (3 x 20 mL). The combined organic layers were washed with saturated aqueous NaCl (40 mL), dried over $MgSO_4$ and the solvent was removed under reduced pressure. The residue was purified by flash column chromatography (hexanes:EtOAc = 9:1) to yield ethyl-5-(((N-Boc-2-aminoethyl)thio)methyl)furan-2-carboxylate (**S8**, 216 mg, 0.960 mmol, 20% yield) as a colourless oil.

R_f = 0.36 [hexanes:EtOAc = 9:1].

^{1}H NMR (400 MHz, $CDCl_3$): δ=7.08 (1 H, d, $^3J_{HH}$ = 3.4 Hz, Fur-*H*), 6.45 (1 H, d, $^3J_{HH}$ = 3.4 Hz, Fur-*H*), 4.90 (1 H, br s, N*H*Boc), 4.32 (2 H, q, $^3J_{HH}$ = 7.1 Hz, C*H*$_2$CH$_3$), 3.73 (2 H, s, Fur-C*H*$_2$-S), 3.27 (2 H, t, $^3J_{HH}$ = 6.1 Hz, SC*H*$_2$CH$_2$), 2.63 (2 H, t, $^3J_{HH}$ = 6.5 Hz, CH$_2$C*H*$_2$NH), 1.40 (9 H, s, C(C*H*$_3$)$_3$), 1.33 (3 H, t, $^3J_{HH}$ = 7.1 Hz, CH$_2$C*H*$_3$) ppm.

^{13}C NMR (101 MHz, $CDCl_3$): δ=158.80, 156.63, 155.95, 144.39, 119.21, 109.89, 79.83, 61.13, 39.56, 32.46, 28.55, 28.13, 14.53 ppm.

**HRMS** (EI):	calcd. for $C_{15}H_{23}NO_5S$	352.1189 $[M + Na]^+$
	found:	352.1184 $[M + Na]^+$.

5-(((*N*-Boc-2-aminoethyl)thio)methyl)furan-2-carboxylic acid (8e)

N-Boc-ethyl-5-(((2-aminoethyl)thio)methyl)furan-2-carboxylate (**S8**, 35.0 mg, 0.106 mmol, 1.0 eq.) was dissolved in MeOH (0.5 mL) and NaOH (4 M in water, 53 mL, 0.212 mmol, 2.0 eq.). The reaction mixture was heated to 60 °C for 4 h; afterwards the solvents were evaporated and the residue was dissolved in EtOAc (5 mL) and washed with water (5 mL) The aqueous layer was extracted with EtOAc and AcOH (3 x 5 mL), the organic layer was dried over Na_2SO_4. All volatiles were removed under reduced pressure to yield 5-(((*N*-Boc-2-aminoethyl)thio)methyl)furan-2-carboxylic acid (**8e**, 28.4 mg, 95.0 µmol, 89% yield).

^{1}H NMR (400 MHz, $CDCl_3$): δ=8.85 (1 H, br s, Fur-COO*H*), 7.21 (1 H, d, $^3J_{HH}$ = 3.4 Hz, Fur-*H*), 6.37 (1 H, d, $^3J_{HH}$ = 3.4 Hz, Fur-*H*), 4.97 (1 H, br s, N*H*Boc), 3.79 (2 H, s, Fur-C*H*$_2$S), 3.34 (2 H, t, $^3J_{HH}$ = 5.8 Hz, SC*H*$_2$CH$_2$), 2.70 (2 H, t, $^3J_{HH}$ = 6.7 Hz, CH$_2$C*H*$_2$NH), 1.46 (9 H, s, -C(C*H*$_3$)$_3$) ppm.

**HRMS** (EI):	calcd. for $C_{13}H_{19}NO_5S$	324.0876 $[M + Na]^+$
	found:	324.0876 $[M + Na]^+$.

Solid phase synthesis

For the synthesis of **chimeras 1-3** and acetylated **controls 1-3**, three myoA tail peptides with different lengths were synthesized on Rink Amide resin on a 40 µmol scale according to previously reported automated Fmoc-based solid phase procedures [J.C. Thomas, J. L. Green, R. I. Howson, P. Simpson, D. K. Moss, S. R. Martin, A. A. Holder, E. Cota and E.W. Tate *Mol. Biosyst. 6* **2010**, 494–498]. The final coupling was to a glycine residue that acted as a spacer between the myoA tail and the fragment functionality. Thus the following sequences were produced (N- to C-):

GLRVQAHIRKKMV-NH_2 **1**

GLLRVQAHIRKKMV-NH_2 **2**

GSLLRVQAHIRKKMV-NH_2 **3**

Each peptide was then either acetylated as reported previously [Thomas *et al.*, 2010, ibid.], or coupled to the fragment functionality using **8e** as detailed below.

For the chimeras, each peptide was placed in a 5 mL syringe with a frit and the resin swelled in DMF (1 mL) by shaking for 1 h at room temperature. Afterwards DMF was removed and reagent mixture (40 µmol **8e**, 40 µmol HATU, 80 µmol DIPEA, 1 mL DMF) was added and the syringe shaken at room temperature for 2 h. The reagent mixture was then removed by filtration. Each resin-bound peptide was then washed with DMF (3 x 5 mL), CH_2Cl_2 (3 x 5 mL), methanol (3 x 5 mL) and diethyl ether (3 x 5 mL) before being dried in a vacuum desiccator for at least 6 h.

The peptides were deprotected and cleaved from the resin by adding 1 mL of a cleavage mixture (94% TFA, 2.5% H_2O, 2.5% (w/v) DTT, 1% TIS) to each resin-bound peptide and shaking at room temperature for 3 h. The liquid was filtered and the resin washed with cleavage mixture (500 µL). Afterwards, to each peptide solution was added 10 mL cold tert-butyl-methyl-ether (TBME) to give a precipitate. This was centrifuged (4000 rpm, 4 °C, 15 min) and the solid washed with TBME (3 x 10 mL). The peptides were dried in a vacuum desiccator overnight before being purified by LC-MS and lyophilized. The characterization data for the peptides are shown in Table S3.

Peptide	Sequence	N-	Calculated mass (Da)	R_t (min)	ES+ peaks (m/z)
**chimera1**	LRVQAHIRKKMV	**8e***	1718	6.82	860 (m/2+1)<br>574 (m/3+1)
**control1**		Ac-	1577	7.05	789 (m/2+1)<br>526 (m/3+1)
**chimera2**	LLRVQAHIRKKMV	**8e***	1831	7.78	917 (m/2+1)<br>611 (m/3+1)
**control2**		Ac-	1690	8.19	845 (m/2+1)<br>564 (m/3+1)
**chimera3**	SLLRVQAHIRKKMV	**8e***	1918	8.05	960 (m/2+1)<br>640 (m/3+1)
**control3**		Ac-	1777	8.40	890 (m/2+1)<br>593 (m/3+1)

Table S3 Characterization data for purified peptides used in this study. All peptides bore C-terminal amides (-NH_2) and were synthesized using Rink Amide AM resin. Analysis of the purified peptides was performed with a Waters LC-MS system using an isocratic gradient of 5-98 % H_2O/MeOH (0.1 % HCOOH) over 18 min. *For the chimeras, **8e** as the N-terminal modification refers to the Boc-deprotected peptide.
